# Prevalence of perennial severe allergic asthma in Italy and effectiveness of omalizumab in its management: PROXIMA – an observational, 2 phase, patient reported outcomes study

**DOI:** 10.1186/s12948-015-0019-7

**Published:** 2015-07-07

**Authors:** Giorgio Walter Canonica, Marta Bartezaghi, Raffaele Marino, Laura Rigoni

**Affiliations:** DIMI, IRCCS AOU San Martino, University of Genoa, Genoa, Italy; Novartis Farma SpA, Largo U. Boccioni 1, Origgio (VA), 21040 Italy

**Keywords:** Omalizumab, IgE, Severe allergic asthma, Perennial, Asthma control

## Abstract

**Background:**

We designed the PROXIMA study (Patient-Reported Outcomes and Xolair^®^ In the Management of Asthma) to determine the proportion of patients with severe asthma sensitive to perennial allergens, and to evaluate asthma control and treatment adherence up to 12 months in patients treated with omalizumab in Italian population. In addition, an ancillary study was designed to explore protein biomarkers and characterize them in relation to severe allergic asthma and treatment effects by proteomic approach.

**Methods:**

PROXIMA is an observational, multicenter, cross-sectional and prospective cohort study conducted at 25 centers in Italy, in outpatient settings. The study consists of two phases: 1) a cross-sectional phase plans to enroll 600 patients with severe allergic asthma, in step 4 therapy as per GINA guidelines, aged ≥18 years, needing a step up in therapy, and 2) a longitudinal phase on patients who will start omalizumab add-on therapy per clinician’s judgment at baseline visit (approximately 180–240 patients). The primary variable of the cross-sectional phase is the proportion of patients with severe asthma presenting with perennial form of allergy (skin prick test or in vitro test). The primary variable of longitudinal phase is proportion of patients who achieve disease control (assessed by Asthma Control Questionnaire [ACQ]) with omalizumab at 6 months, and maintain it at 12 months. Secondary variables are patient compliance to omalizumab, patient-reported perception of cognitive and emotional impact of the illness, assessed by Brief Illness Perception Questionnaire (Brief IPQ) and the health related quality of life evaluated by the EuroQoL 5D-3 L (EQ-5D-3 L). Safety endpoints will be recorded during the course of the study. Patients participating in the longitudinal phase will be enrolled for ancillary study if they provide additional informed consent. Protein species in complex mixtures will be identified using innovative MudPIT (Multidimensional Protein Identification Technology) method.

**Conclusions:**

The results of this observational study will provide estimate of patient population allergic to perennial allergens in Italy and information on patient-reported outcomes with omalizumab therapy in a real-world setting. The exploratory proteomic analysis on asthma biomarkers could eventually provide new data to identify responder patients to anti IgE therapy.

## Background

Asthma is a highly prevalent respiratory disorder characterized by chronic inflammation and hyper-responsiveness of airways, and variable airflow obstruction, often reversible [1]. More than 300 million people are affected by asthma, worldwide [1,2]. Of this population, about 10% is estimated to have severe asthma accounting for significant burden of morbidity and mortality [3,4]. In Italian population (GEIRD Study), the median prevalence of asthma was reported to be 6.6%, ranging from 4.5%–8.5%, recording a 35% increase in the last 2 decades [5]. Clinically, many different forms or phenotypes of asthma have been recognized; however, allergic asthma affects a substantial proportion of patients with asthma [2]. Atopy and allergic mechanisms have been implicated for asthma in 50-80% of the patients and in approximately 50% of patients with severe asthma [6-8]. In Italy, allergic asthma affects >60% patients with asthma [5].

In patients with allergic asthma, who are already sensitized to a specific environmental allergen, exposure to same allergen acts as powerful trigger for asthmatic response, which is mast-cell driven in the early phase and inflammatory-cell driven in late phase [1,8]. Markedly increased levels of IgE antibodies are found in individuals with allergic asthma. IgE antibodies cause chronic airway inflammation via high-affinity (FcεRI) or low-affinity (FcεRII) IgE receptors present on effector cells such as mast cells, and basophils [1,8]. Antigen induced cross-bridging of IgE-FcεRI cell surface complexes, triggering degranulation of effector cells, gives rise to various symptoms of allergy. The distinction between perennial and seasonal allergic asthma are often made on the basis of the seasonality of sensitizing allergens (seasonal and perennial) and the detection IgE specific to allergen [8]. However, distinction between seasonal and perennial allergic asthma based on seasonality of allergens has been a topic for discussion among clinicians because of certain overlapping characteristics of allergens. Recently, it was proposed to replace the terms seasonal and perennial with the ‘intermittent’ and ‘persistent’ - disease or -exposure to allergens. The concept was intended to introduce change in the treatment approach for allergic diseases taking into account the overlap between the treatment of seasonal and perennial allergies. The new definition was proposed because the concept of seasonality could not be applied to some of the perennial allergens (occupational, mite and pet allergens) and some seasonal environmental allergens (pollen) might be considered as perennial owing changes in climate. Moreover, the majority of patients are usually sensitized with multiple allergens (polysensitization) with occupational allergens, mite and pet allergens, and presence of cross-reactivity and pan-allergens [9].

Omalizumab, a recombinant monoclonal antibody (IgE antibody), reduces free IgE levels by binding to the Fc domain of the circulating IgE and thereby inhibiting IgE binding to FcεRI receptors. In addition omalizumab also blocks the IgE-dependent uptake of allergens by mature myeloid dendritic cells, thereby affecting the chronic allergic response [7,10]. Currently, omalizumab is the recommended treatment of severe allergic asthma in patients not responding to Step 4 of the GINA treatment approach, and need therapeutic step up to Step 5 [11]. In the European Union, omalizumab is approved as add-on therapy in adults, adolescents, and children (6 to <12 years of age) with severe persistent allergic asthma inadequately-controlled (reduced lung function, frequent daytime symptoms or night-time awakenings, and multiple documented severe asthma exacerbations), despite daily high-dose inhaled corticosteroids (ICS), plus a long-acting inhaled beta2-agonist (LABA) [12].

Medical literature lacks information on the proportion of patients with severe asthma presenting with the perennial form of allergy in Italy. Moreover, data on the effectiveness of omalizumab as add-on therapy for patients with severe persistent allergic asthma in the Italian population is limited.

### Primary objectives

The PROXIMA (Patient Reported Outcomes and Xolair^®^ In the Management of Asthma) study was designed to estimate the prevalence of perennial allergy in adult patients with severe allergic asthma in the Italian population and to evaluate the proportion of patients in this demographics who achieve disease control at 6 months and maintain it at 12 months in a sample of patients treated with omalizumab.

## Methods

### Study design and patient population

**Figure 1:**
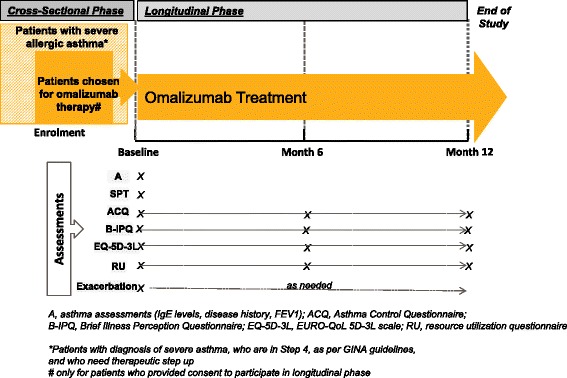
Study design and assessments.

PROXIMA is an observational, multicenter, cross-sectional and prospective cohort study conducted at 25 centers in Italy, in outpatient settings (Hospitals and University centers specialized in the treatment of asthma). The study comprises of 2 phases, a cross-sectional and a longitudinal phase (12 months). Patients entered in the longitudinal phase will be treated with omalizumab as per “scheda tecnica” [12] (Figure 1).

In the cross-sectional phase, about 600 patients aged ≥18 years, diagnosed with severe allergic asthma currently in Step 4 (as defined by GINA international guidelines) and need a step up in therapy will be enrolled. About 30%–40% (180–240 patients with perennial allergic asthma) are estimated to enter the longitudinal phase, start therapy with omalizumab, followed up for 12 months. Omalizumab as treatment choice will be based on clinician’s judgment and local treatment guidelines. As the study will involve questionnaire based assessments, patient unable to complete the patient questionnaires will not be enrolled for the study. Patients participating in the longitudinal phase will have the option to participate to an ancillary study (The PROXIMA sub-study: exploratory proteomic analysis on asthma biomarkers).

This study was designed and shall be implemented and reported in accordance with the Guidelines for Good Pharmacoepidemiology Practices (GPP) of the International Society for Pharmacoepidemiology (ISPE 2008), the STROBE (Strengthening the Reporting of Observational Studies in Epidemiology) guidelines, the ethical principles laid down in the Declaration of Helsinki, and the AIFA (Agenzia Italiana del Farmaco) guideline for the classification and management of observational studies on drugs [13-15].

Eligible patients may be enrolled only after written informed consent. The study documents including protocol and informed consent forms were approved by the ethics committees and institutional review boards of all participating centers.

### Variables

The two primary variables of the study are the proportion of patients with severe allergic asthma having allergy to a perennial form of aeroallergens (at baseline cross-sectional phase) and the proportion of patients with asthma control at Months 6 and 12 (in longitudinal phase) with omalizumab therapy. Secondary variables in the cross-sectional phase are the level of asthma control, patient disease perception at baseline in all patients (whole sample) and in patients with perennial allergy compared with seasonal allergy, quality of life and healthcare resource utilization. In the longitudinal phase secondary variables are, 1) proportion of patients with at least 1 episode of asthma exacerbation during the 12 months study period; 2) patient compliance to omalizumab at 6 and 12 months; 3) persistence with omalizumab treatment for 12 month; and 4) quality of life and healthcare resource utilization at months 6 and 12.

### Assessments

Patients with perennial form of allergy will be identified by clinicians on the basis of the results of skin prick test or in vitro tests for the reactivity to a perennial aeroallergen. Asthma disease control will be assessed by the Asthma Control Questionnaire (ACQ) using a 7-point scale, where 0 indicates well-controlled and 6 signify extremely poorly controlled asthma [16]. A patient will be defined (as per protocol) as controlled if the ACQ total score will be between 0 and 4.

Brief Illness Perception Questionnaire (Brief IPQ) and EuroQoL 5D-3 L (EQ-5D-3 L) scale will be used to assess patient disease perception and quality of life, respectively. Brief IPQ is a 9-item questionnaire designed to rapidly assess cognitive and emotional representations of illness [17]. All of the questionnaire items (except the causal question, item 9) are rated using a 0 to-10 response scale; five of the items assess cognitive illness representations, two of the items assess emotional representations and one item assesses illness comprehensibility. Assessment of the causal representation is by an open response item, which asks patients to list the three most important causal factors in their illness. EQ-5D-3 L scale consists of two sections, EQ-5D-descriptive system and the EQ-visual analogue scale (EQ-VAS) [18,19]. The EQ-5D-3 L descriptive system assesses 5 dimensions: mobility, self-care, usual activities, pain/discomfort and anxiety/depression. Each dimension has 3 levels: no problems, some problems, extreme problems. EQ-VAS is used as a quantitative measure of health as judged by the individual respondents on a visual analogue scale from 0 to 100, where 0 the “worst imaginable health state” and 100 is the “best imaginable health state”.

Patient compliance will be assessed as the ratio between the number of injections of omalizumab administered during the observational period (month 6 and 12) over the total number of planned injections. Omalizumab persistence during the 12 months will be assessed by Kaplan-Meier survival curve analysis, where the event of interest will be the treatment discontinuation.

The consumption of healthcare resources will be assessed by using a dedicated questionnaire collecting information on the number of hospitalizations, number of emergency department visits, number of outpatient visits and number of laboratory and diagnostic tests.

Asthma exacerbations, serum IgE levels and forced expiratory volume in 1 s (FEV_1_) percent (at baseline) will be measured by using standard clinical and laboratory assessment methods [20]. Safety assessments consist of clinical examination, monitoring and recording all adverse events and serious adverse events. All events will be reported to local Health Authorities according to Italian regulatory requirements and recorded in the Novartis safety database.

### Statistical considerations

The primary variable in the cross-sectional phase the proportion of patients with a perennial form of aeroallergens will be calculated as the ratio of the number of patients with a perennial form of aeroallergens to the total number of evaluable patients with a diagnosis of severe allergic asthma at baseline.

The primary variable in the longitudinal phase, the proportion of controlled patients based on ACQ both at 6 months and at 12 months, will be calculated as the ratio between the patients who are controlled and the total number of evaluable patients treated with omalizumab. Both of the primary variables will be presented with 95% confidence limit. The proportion of patients who change status from controlled to un-controlled and vice-versa between 6 and 12 months will also be presented.

With respect to the longitudinal phase, descriptive statistics for each time point as well as changes versus baseline will be provided for the ACQ total score, brief IPQ and EQ-5D-3 L. The patient compliance with omalizumab use will be evaluated, as the ratio between the numbers of administered injections during the observational period over the total number of planned injections, at 6 and 12 months after treatment start.

### Sample size estimations

The sample size was determined on the basis of feasibility criteria. According to the volume of patients managed by the centers involved in this study, it is reasonable to suppose the inclusion of 600 eligible outpatients are adequate to arrive at sufficient sample size for the study. Considering that 5% patients may not be available for analysis, because of missing data or inclusion–exclusion criteria violations, a total of 570 evaluable patients, allows a precision of estimate between 2.5% and 3.9% of the proportion of patients with a perennial form of aeroallergens. In particular the two sided 95% confidence interval will be 7.5%–12.5% for the 10% expected proportion of patients and 31.1%-38.9% for the 35% expected proportion, based on the results of ENFUMOSA study [3]. The proportion of patients starting omalizumab at baseline is expected to range between 30%–40%. Therefore, the sample size of patients to be included in the longitudinal phase should range from 180–240 patients. Assuming a 12 months drop-out rate (including non-evaluable patients) of about 15%, 153–204 patients will be evaluable for the primary endpoint in the longitudinal phase. Moreover, based on the response of the omalizumab treatment as assessed by using global evaluation of treatment effectiveness (GETE) tool, it was appropriate to assume that the proportion of patients with disease control after 12 months will range from 50%–70%. A sample of 153 and 204 evaluable patients allows a precision of estimate between 7.9%–7.3% and 6.9%–6.3%, for the proportion of patients who achieve the disease control at month 6 and maintains it at month 12 [21,22].

### The PROXIMA ancillary-study: exploratory proteomic analysis on asthma biomarker

#### Rationale

Asthma is a complex respiratory disorder known to have multiple causes, phenotypes, and treatment responses. However, this complexity provides an opportunity for personalized and targeted therapy by characterizing specific biomarkers aiding to early diagnosis, assessing therapeutic responses and even validation for new drug targets [23]. Recent advances in mass spectrometry-based proteomic approaches may facilitate identification and characterization of new biomarkers in asthma and subsequently improve understanding of their clinical significance [24,25].

Currently, biomarkers that have known diagnostic and clinical role in asthma are poorly characterized and there are no proteomic-based studies to describe inflammatory biomarkers during and after the treatment of severe asthma.

#### Objectives

This ancillary study was designed to explore protein biomarkers in patients with severe allergic asthma treated with omalizumab and characterize potential biomarkers in relation to the achievement and maintenance of disease control. In addition, the study aims to identify proteotypical peptides useful for the development of diagnostic methods.

#### Study design and patient population

The PROXIMA ancillary study is planned to be conducted at 8 centers in Italy with the aim of characterizing biomarkers related to severe allergic asthma by a proteomic approach. The patients who would participate in the longitudinal phase of PROXIMA study and provide additional informed consent specific to the ancillary study will be enrolled.

#### Sample collections and investigations

Three samples for blood and urine will be collected from eligible patients at baseline, 6 and 12 months of the PROXIMA main study. The samples will be collected, stored and shipped, as per instructions defined by the central laboratory, to conduct the proteomic analysis at Internal Medicine Department at Respiratory Diseases Clinic, Hospital San Martino, University of Genoa.

A gel-free system and mass spectrometry will be used to explore the proteins biomarkers. Protein species in complex mixtures will be identified using innovative MudPIT (Multidimensional Protein Identification Technology) method [26]. The MudPIT method consists of a two-dimensional chromatography coupled to the mass spectrometry in tandem (2 DC-MS/MS) that allows the identification of thousands of proteins simultaneously from single complex sample [27].

#### Statistical analysis

There was no formal statistical hypothesis was set for the study. Descriptive analyses will be performed. The list and frequency of all proteins identified in the total sample of patients will be provided at baseline, 6 and 12 months of omalizumab treatment. In order to assess whether asthma protein biomarkers are related to the achievement and maintenance of disease control, the frequency of all identified proteins will be stratified by responders and non-responders (as defined in the PROXIMA main study). Since a large number of possible proteins are expected to be observed, Bonferroni’s correction will be applied. The 95% confidence interval will be calculated for both objectives.

## Discussion and conclusions

The PROXIMA observational study is designed to provide estimates on the number of patients with asthma who are allergic to perennial allergens and to assess the effectiveness of omalizumab in patients with severe allergic asthma in the Italian population for the first time. In various clinical studies, omalizumab has been shown to improve asthma control and reduce exacerbations in patients with inadequately-controlled severe allergic asthma [4]. Current study is planned to be conducted in a real-world setting in Italy and the result will further confirm the control of asthma achieved and maintained by omalizumab therapy in this population. The exploratory proteomic analysis on asthma biomarkers could identify proteins of interest in patients with asthma, and link them to the patients’ level of response to the disease and treatment. The new data that will help to identify and characterize the best responders to omalizumab treatment will be a step towards personalized medicine.

## Abbreviations

2 DC-MS/MS: Two-dimensional chromatography coupled to the mass spectrometry in tandem
ACQ: Asthma Control Questionnaire
AIFA: Agenzia Italiana del Farmaco
Brief IPQ: Brief Illness Perception Questionnaire
EQ VAS: EQ-visual analogue scale
EQ-5D-3 L: EuroQoL 5D-3 L
FEV_1_: Forced expiratory volume in 1 s
GETE: Global evaluation of treatment effectiveness
GINA: Global Initiative for Asthma
GPP: Good Pharmacoepidemiology Practices
ICS: Inhaled corticosteroids
IgE: Immunoglobulins E
ISPE: International Society for Pharmacoepidemiology
LABA: Long-acting inhaled beta2-agonist
MudPIT: Multidimensional Protein Identification Technology
PROXIMA: Patient Reported Outcomes and Xolair^®^ In the Management of Asthma
STROBE: Strengthening the Reporting of Observational Studies in Epidemiology
